# Prevalence of metabolic syndrome among adults with liver function injury in rural area of Southwest China: A cross-sectional study

**DOI:** 10.1038/s41598-017-05791-2

**Published:** 2017-07-17

**Authors:** Hui Zeng, Hui Lin, Wenyi Liu, Jia Wang, Lingqiao Wang, Chuanfen Zheng, Yao Tan, Yujing Huang, Lixiong He, Jiaohua Luo, Chaowen Pu, Renping Zhang, Xiaohong Yang, Yingqiao Tian, Zhiqun Qiu, Ji-an Chen, Yang Luo, Xiaobin Feng, Guosheng Xiao, Liping Wu, Weiqun Shu

**Affiliations:** 1 Department of Environmental Hygiene, College of Preventive Medicine, Third Military Medical University, Chongqing, China; 2 Department of Tropical Epidemiology, College of Preventive Medicine, Third Military Medical University, Chongqing, China; 3 The Center for Disease Control and Prevention in Fuling District, Chongqing, China; 4 Department of Health Education, College of Preventive Medicine, Third Military Medical University, Chongqing, China; 5 Center for Nanomedicine, Southwest Hospital, Third Military Medical University, Chongqing, China; 6 Institute of Hepatobiliary Surgery, Southwest Hospital, Third Military Medical University, Chongqing, China; 7 College of Life Science and Engineering, Chongqing Three Gorges University, Wanzhou, Chongqing China

## Abstract

Abnormal liver function (ALF) plays a key role in metabolic syndrome (MetS), but only few data on the relationship between MetS and the risk factors for ALF (e.g., biotoxins) are available. We aimed to provide the prevalence of MetS and its association with the risk factors for ALF in rural area of Southwest China. A cross-sectional study within the hepatocellular carcinoma cohort was conducted, and included 5493 people with age from 30 to 85 years old. MetS was defined according to the Joint Scientific Statement. We observed that the prevalence of MetS was 31.8% (39.0% in women and 19.8% in men). Logistic regression analysis showed that significantly increased risk of MetS was found in those showing ALF (OR = 3.00, 95% CI: 2.43–3.71). Significantly decreased risk of MetS was found in those with higher HBV DNA titers (OR = 0.49, 95% CI: 0.33–0.74), and in those with higher aflatoxin B1 exposure (estimated daily intake, EDI) (OR = 0.60, 95% CI: 0.53–0.67). No significant change was found in those with higher microcystin-LR exposure (EDI). Therefore, the different risk factors for ALF might exert different effects on MetS. However, there should be an interaction effect existing that might decide the severity of MetS.

## Introduction

The metabolic syndrome (MetS) is defined as a well-recognized clinical complex disorder characterized by elevated fasting glucose (GLU, ≥100 mg/dL), elevated blood pressure (BP, Systolic ≥130 and/or diastolic ≥85 mm Hg), reduced high-density lipoprotein cholesterol (HDL-C, <40 mg/dL in males; <50 mg/dL in females), elevated triglycerides (TG, ≥150 mg/dL) and elevated waist circumference (WC), according to the Joint Scientific Statement “Harmonizing the Metabolic Syndrome” published in *Circulation* on October 20^th^, 2009^1^. Several previous research studies reported that MetS can increase risks of cardiovascular disease threefold^2^, risks of type 2 diabetes two- to fivefold^3^, risks of myocardial infraction three- to fourfold, and risks of mortality twofold^4^. The prevalence of MetS is ranging from <10% to 84% worldwide^5–8^. It is 34.3% among all adults, 36.1% among men, and 32.4% among women in US^7^; In china, MetS has also become a significant burden to the society. A higher prevalence of MetS (33.9% among all people aged >32 years old, 32.5% among women and 35.1% among men) has been reported in urban community (Zhabei District of Shanghai)^9^. A meta-analysis also showed that the prevalence in China is 24.5% (19.2% in males and 27.0% in females) among subjects aged 15 years and older^10^ despite of the difference in diagnose criteria for MetS.

MetS is known to be positively associated with different types of chronic liver diseases, notably nonalcoholic fatty liver disease (NAFLD)^11–13^. However, the pathophysiology of MetS is still unclear. As we know, the liver plays an essential role in metabolism of carbohydrate, protein and lipid, and then might also play a key role in the development of metabolic syndrome. Therefore, the risk of abnormal liver function (ALF) might also play a key role in the progression of MetS. Hepatitis B virus (HBV) infection, aflatoxin B1 (AFB1) exposure and microcystin-LR (MC-LR) exposure are the known environmental risk factors for ALF and liver disease^14, 15^. However, previous research gives conflicting results on the association between MetS and the risk factors for ALF. For example, some researchers found no associations^16^ between MetS and HBV infection, some found a positive association^17^, and others found an inverse association^18, 19^. As yet there has been little research reporting the association between MetS and AFB1/MC-LR exposure. Therefore, the relationship between MetS and the risk factors for ALF is still largely unknown.

We used the data from a cross-sectional study on hepatocellular carcinoma in the Three Gorge Reservoir of China in 2013 to estimate the prevalence of MetS in the adult population in the rural area of Southwest China, where there is a higher prevalence of HBV infection and a wide contamination of AFB1 and MC-LR as we previously reported^20^, to describe the association between the risk factor for ALF and MetS, and to analyze the modification effects of liver function injury.

## Results

### The prevalence of MetS and its constituents

**Figure 1:**
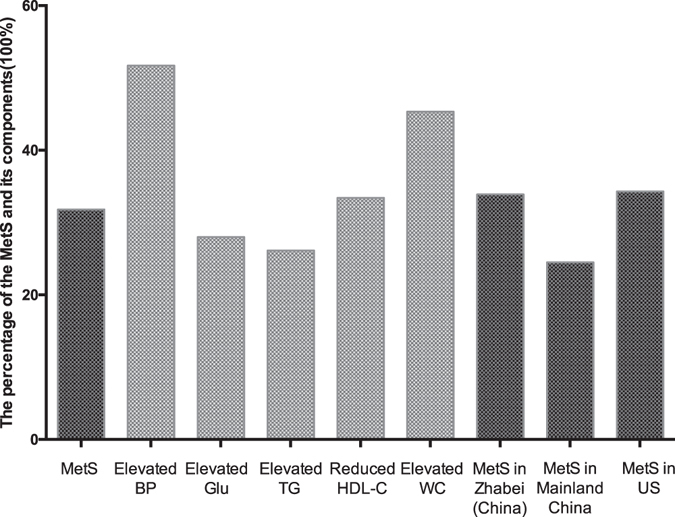
The prevalence of the MetS and its constituents in rural area located in Southwest China. We observed that the prevalence of MetS (31.8%) was lower than the prevalence level in Zhabei District of Shanghai in China (33.9%) reported in 2013 and that in US (34.3%) reported in 2010, but higher than that in mainland China (24.5%) reported in 2016.

Of all the 5493 participants, 1747 subjects (31.8%) had MetS, among which, 39.0% were women and 19.8% were men. 2839 subjects (51.7%) showed elevated BP, 538 subjects (28.0%) elevated GLU, 1436 subjects (26.1%) elevated TG, 1836 subjects (33.4%) reduced HDL, and 2490 subjects (45.3%) elevated WC (Fig. 1).

### The characteristics of the subjects with MetS

People with MetS were older than the healthy ones (60.21 + 11.83 y *versus* 58.09 + 12.83 y). The percentage of people with HBV infection was 8.2%, that of people with cigarette smoking 30.8%, that of people with alcohol drinking 23.6%, that of people with ALF 8.5%, that of people with abnormal salt consumption 65.0%, that of people with junior high school education level 65.3%, and that of farmers (living in the village) 51.2%, respectively (data not shown).

Among them, we observed that the percentage of MetS increased, from 19.8% to 36.9% in subjects with advancing age, from 27.5% in those never consuming fat to 37.6% in those consuming fat more than once a week, from 30.0% in those without ALF to 51.0% in those with ALF, from 29.9% in those without labor strength to 37.5% in those with lower labor strength, and from 31.4% living in small towns to 33.1% living in villages. We also observed a lower percentage of MetS, 23.0% in people with positive HBsAg (*versus* 32.5% in those without), 26.3% in people with higher AFB1 exposure (*versus* 37.3% in those with lower), 30.9% in people with higher MC-LR exposure (*versus* 32.7% in those with lower), 18.8% in those with ever cigarette smoking (*versus* 35.8% in those without), 22.7% in those with alcohol drinking (*versus* 34.0% in those without), 27.0% in those with heavy labor strength (*versus* 29.9% in those with low labor strength), and 26.8% in those of junior high education (*versus* 34.5% in those of primary education). However, there was no significant difference in percentage of MetS among people having different salt, fruit and vegetable consumption levels, and family income levels, although some of the factors are known to be the protective or risk factors for MetS (Table 1).

**Table 1:** Sociodemographic characteristics of subjects with and without MetS in rural areas located in Southwest China, 2013 (n = 5493).

Demographic variables		Normal N (%)	MetS N (%)	Adjusted OR^a^ (95% CI)	p
Total		3746	1747		
Area	Small town	1865 (69.6)	816 (31.4)	1.00 (Reference)	
	Village	1881 (66.9)	931 (33.1)	1.33 (1.15–1.53)	<0.001
Sex	Male	1657 (80.2)	410 (19.8)	1.00 (Reference)	
	Female	2089 (61.0)	1337 (39.0)	2.48 (2.05–2.99)	<0.001
Age (y)	~40	326 (80.3)	80 (19.8)	1.00 (Reference)	
	41~50	1080 (71.7)	426 (28.3)	1.64 (1.24–2.17)	0.001
	51~60	558 (67.8)	265 (32.2)	2.07 (1.53–2.80)	<0.001
	61~70	1084 (63.1)	635 (36.9)	2.55 (1.91–3.40)	<0.001
	71~	698 (67.2)	341 (32.8)	2.23 (1.64–3.04)	<0.001
HBsAg	Negative	3418 (67.5)	1649 (32.5)	1.00 (Reference)	
	Positive	322 (77.0)	96 (23.0)	0.60 (0.46–0.77)	<0.001
AFB_1_	Under median (<7.70 ng/day/kg)	1722 (62.7)	1024 (37.3)		
	Above median (≥7.70 ng/day/kg)	2024 (73.7)	723 (26.3)	0.60 (0.53–0.67)	<0.001
MC-LR	Under median (<3.17 ng/day/kg)	1847 (67.3)	898 (32.7)		
	Above median (≥3.17 ng/day/kg)	1899 (69.1)	849 (30.9)	0.89 (0.77–1.02)	0.10
Cigarette Smoking	Never	2697 (64.2)	1504 (35.8)	1.00 (Reference)	
	Ever	1049 (81.2)	243 (18.8)	0.79 (0.63–0.99)	0.04
Alcohol drinking	Never	2935 (66.0)	1509 (34.0)	1.00 (Reference)	
	Ever	811 (77.3)	238 (22.7)	1.14 (0.94–1.40)	0.19
Fat consumption	Never	1678 (72.5)	635 (27.5)	1.00 (Reference)	
	Current, once a week	1099 (67.3)	533 (32.7)	1.03 (0.89–1.20)	0.71
	Current, above once a week	945 (62.4)	570 (37.6)	1.20 (1.03–1.40)	0.02
Salt consumption	<6 g/day	1300 (67.8)	618 (32.2)	1.00 (Reference)	
	6~12 g/day^b^	1760 (68.4)	814 (31.6)	0.97 (0.85–1.12)	0.75
	>12 g/day	683 (68.6)	313 (31.4)	0.97 (0.81–1.17)	0.75
Fresh food and vegetable intake	<500 g/day	726 (66.9)	359 (33.1)	1.09 (0.93–1.28)	0.29
	500~900 g/day^b^	2049 (68.9)	923 (31.1)		
	>900 g/day	936 (67.5)	450 (32.5)	1.10 (0.95–1.28)	0.19
Liver function	Normal	3518 (70.0)	1510 (30.0)	1.00 (Reference)	
	Abnormal	228 (49.0)	237 (51.0)	3.00 (2.43–3.71)	<0.001
Labor strength	Appropriate	1855 (70.1)	793 (29.9)	1.00 (Reference)	
	Low	1078 (62.5)	647 (37.5)	1.23 (1.07–1.41)	0.004
	Heavy	790 (73.0)	292 (27.0)	0.91 (0.76–1.07)	0.25
Family Income	<6 000 RMB/year	1606 (68.3)	744 (31.7)	0.99 (0.86–1.14)	0.89
	6 000~12 000 RMB/year^c^	1480 (68.3)	688 (31.7)	1.00 (Reference)	
	>12 000 RMB/year	623 (67.8)	296 (32.2)	1.16 (0.97–1.39)	0.11
Education level	Primary school	2351 (65.5)	1236 (34.5)	1.00 (Reference)	
	Junior high school	1395 (73.2)	511 (26.8)	0.89 (0.77–1.04)	0.13

^a^The adjusted ORs were estimated by a binary logistic regression model that included sex, age, HBV infection, AFB1 exposure (EDI), MC-LR exposure (EDI), cigarette smoking, alcohol drinking, fat consumption, salt consumption, liver function, labor strength, family income and education level, except for the factors that had potential modification effects on MetS; ^b^Recommended daily intake; ^c^Average family income level.

Binary logistic regression analyses showed that significantly increased risk of MetS was found in females (OR = 2.48, 95% CI: 2.05–2.99, compared with males), in those with advanced age (OR = 1.64, 95% CI: 1.24–2,17), in those consuming fat more than once a week (OR = 1.20, 95% CI: 1.03–1.40, compared with those without fat consumption), in those showing ALF (OR = 3.00, 95% CI: 2.43–3.71, compared with those showing normal liver function), in those with lower labor strength (OR = 1.23, 95% CI: 1.07–1.41, compared with those with proper labor strength), and in those living in villages (OR = 1.33, 95% CI: 1.15–1.53, compared with those living in small towns) after adjusted for other potential risk factors. The protective factors for MetS were HBV infection (OR = 0.60, 95% CI: 0.46–0.77), the higher AFB1 exposure (OR = 0.60, 95% CI: 0.53–0.67) and cigarette smoking (OR = 0.79, 95% CI: 0.63–0.99) (Table 1).

### The association between liver function and MetS

**Figure 2:**
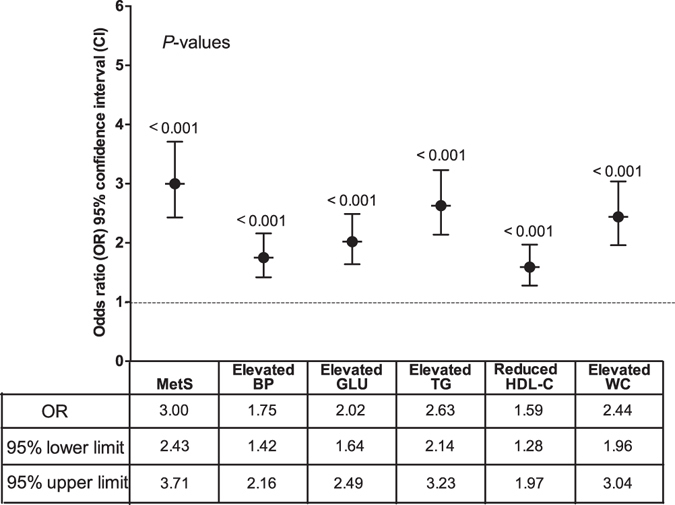
Association between abnormal liver function and MetS in rural areas located in Southwest China. Abnormal liver function increased the risk of MetS (OR = 3.00, 95% CI: 2.43–3.71) and all its constituents after adjustment for age, sex, HBV infection, Aflatoxin B1 exposure, MC-LR exposure, alcohol drinking, cigarette smoking, labor strength, education level, family income, and fat, salt, vegetable and fruit consumption.

Figure 2 shows that ALF significantly increased the risk of MetS (OR = 3.00, 95% CI: 2.43–3.71), and also increased the risk of its constituents: elevated BP (OR = 1.75, 95% CI: 1.42–2.16), elevated GLU (OR = 2.02, 95% CI: 1.64–2.49), elevated TG (OR = 2.63, 95% CI: 2.14–3.23), reduced HDL-C (OR = 1.59, 95% CI: 1.28–1.97) and elevated WC (OR = 2.44, 95% CI: 1.96–3.04).

### The association between HBV infection and MetS

**Figure 3:**
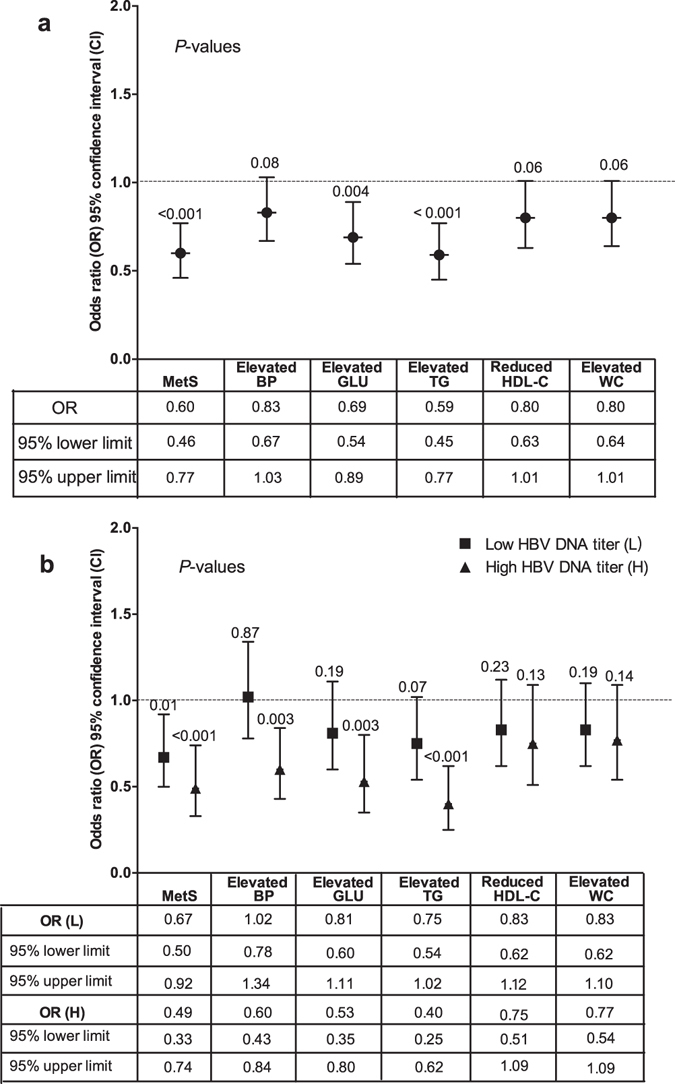
Association between hepatitis B virus (HBV) infection and MetS in rural areas located in Southwest China. (**a**) Association between HBsAg positive and MetS. HbsAg positive decreased the risk of MetS (OR = 0.60, 95% CI: 0.46–0.77), elevated GLU (OR = 0.69, 95% CI: 0.54–0.89) and elevated TG (OR = 0.59, 95% CI: 0.45–0.77). (**b**) Association between HBV DNA titers and MetS. Lower HBV DNA titers decreased the risk of MetS (OR = 0.67, 95% CI: 0.50–0.92), but did not decrease its constituents; higher HBV DNA titers decreased the risk of MetS (OR = 0.49, 95% CI: 0.33–0.74) and its three constituents. All the results were analyzed after adjustment for age, sex, abnormal liver function, Aflatoxin B1 exposure, MC-LR exposure, alcohol drinking, cigarette smoking, labor strength, education level, family income, and fat, salt, vegetable and fruit consumption.

Figure 3a shows that HBV infection decreased the risk of MetS (OR = 0.60, 95% CI: 0.46–0.77). In all constituents of MetS, HBV infection also significantly decreased the risk of elevated fasting glucose (OR = 0.69, 95% CI: 0.54–0.89) and elevated TG (OR = 0.59, 95% CI: 0.45–0.77), respectively. Figure 3b shows that low HBV DNA titers significantly decreased the risk of MetS (OR = 0.67, 95% CI: 0.50–0.92) but did not obviously decreased the risk of the constituents of MetS; higher HBV DNA titers obviously decreased not only the risk of MetS (OR = 0.49, 95% CI: 0.33–0.74) but also the risk of some constituents of MetS: elevated BP (OR = 0.60, 95% CI: 0.43–0.84), elevated GLU (OR = 0.53, 95% CI: 0.35–0.80) and elevated TG (OR = 0.40, 95% CI: 0.25–0.62).

### The association between AFB1 exposure (estimated daily intake, EDI) and MetS

**Figure 4:**
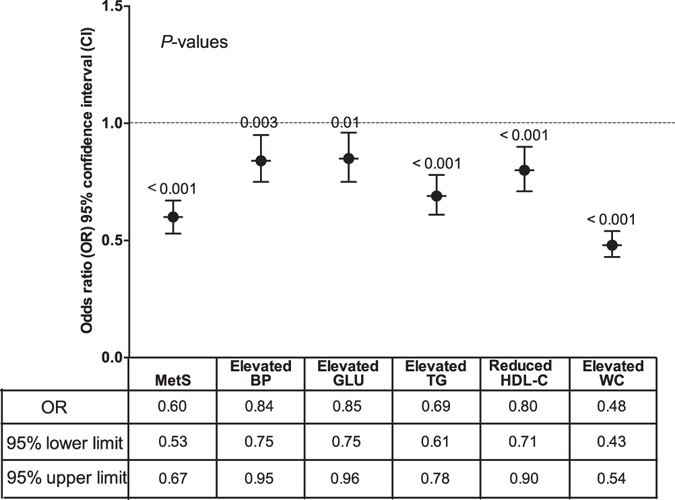
Association between Aflatoxin B1 (AFB1) and MetS in rural areas located in Southwest China. Higher AFB1 exposure decreased the risk of MetS (OR = 0.60, 95% CI: 0.53–0.67) and all its constituents after adjustment for age, sex, HBV infection, MC-LR exposure, alcohol drinking, cigarette smoking, labor strength, education level, family income, and fat, salt, vegetable and fruit consumption.

Figure 4 shows that the higher AFB1 exposure (EDI) significantly decreased the risk of MetS (OR = 0.60, 95% CI: 0.53–0.67), and also decreased the risk of all its constituents: elevated BP (OR = 0.84, 95% CI: 0.75–0.95), elevated GLU (OR = 0.85, 95% CI: 0.75–0.96), elevated TG (OR = 0.69, 95% CI: 0.61–0.78), reduced HDL-C (OR = 0.80, 95% CI: 0.71–0.90) and elevated WC (OR = 0.48, 95% CI: 0.43–0.54).

### The association between MC-LR exposure (EDI) and MetS

There was no significant association between the MC-LR exposure (EDI) and MetS (data not shown).

### The modification effects of liver function, HBV infection, AFB1 exposure and MC-LR exposure on MetS and its constituents

**Figure 5:**
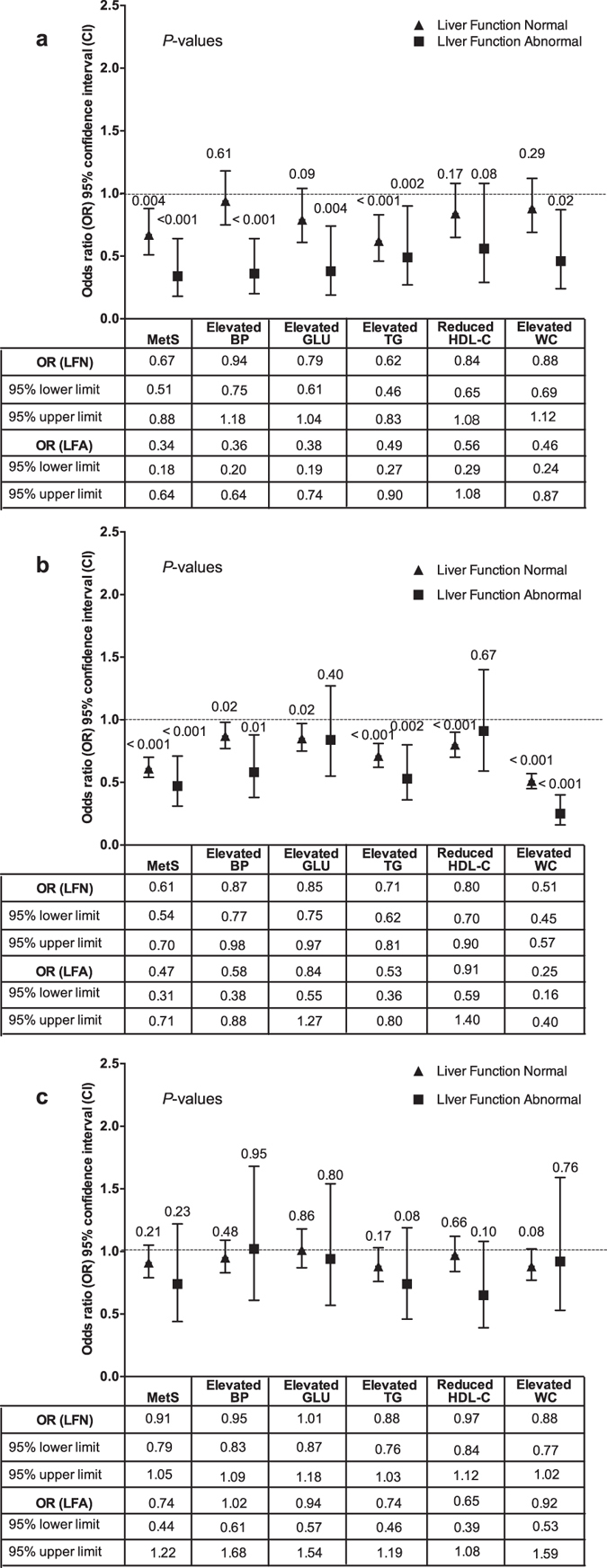
The modification effects of liver function on relationships of MetS and its constituents with HBV infection, Aflatoxin B1 exposure and MC-LR exposure. (**a**) The association between the liver function and HBV infection. (**b**) The association between the liver function and Aflatoxin B1 exposure. (**c**) The association between the liver function and MC-LR exposure. HBV infection and the higher AFB1 exposure (EDI) were inversely associated with MetS no matter whether the liver function was normal or abnormal. For people with HBV infection, the number of the constituents of MetS with decreased risk was higher in those showing abnormal liver function than in those showing normal liver function. For people with the higher AFB1 level, the number of the constituents of MetS with decreased risk were lower in those showing abnormal liver function than in those showing normal liver function. For people showing either normal or abnormal liver function, those exposed to the higher or lower MC-LR levels showed no difference in the risk of MetS and all its constituents.

For people showing normal liver function, those detected HbsAg positive had the obviously decreased risk of MetS (OR = 0.67, 95% CI: 0.51–0.88) and elevated TG (OR = 0.62, 95% CI: 0.46–0.83), compared with those detected HbsAg negative; for people showing abnormal liver function, those detected HbsAg positive had the obviously decreased risk of MetS (OR = 0.34, 95% CI: 0.18–0.64), elevated BP (OR = 0.36, 95% CI: 0.20–0.64), elevated GLU (OR = 0.38, 95% CI: 0.19–0.74), elevated TG (OR = 0.49, 95% CI: 0.27–0.90) and elevated WC (OR = 0.46, 95% CI: 0.24–0.87), compared with those detected HbsAg negative (Fig. 5a). For people showing normal liver function, those exposed to the higher AFB1 levels had the obviously decreased risk of MetS (OR = 0.61, 95% CI: 0.54–0.70) and all its constituents, compared with those exposed to the lower AFB1 levels; for people showing abnormal liver function, those exposed to the higher AFB1 levels had the obviously decreased risk of MetS (OR = 0.47, 95% CI: 0.31–0.71), and elevated BP (OR = 0.58, 95% CI: 0.38–0.88), elevated TG (OR = 0.53, 95% CI: 0.36–0.80) and elevated WC (OR = 0.25, 95% CI: 0.16–0.40), compared with those exposed to the lower AFB1 levels (Fig. 5b). For people showing either normal or abnormal liver function, those exposed to the higher or lower MC-LR levels showed no difference in the risk of MetS and all its constituents (Fig. 5c).

**Figure 6:**
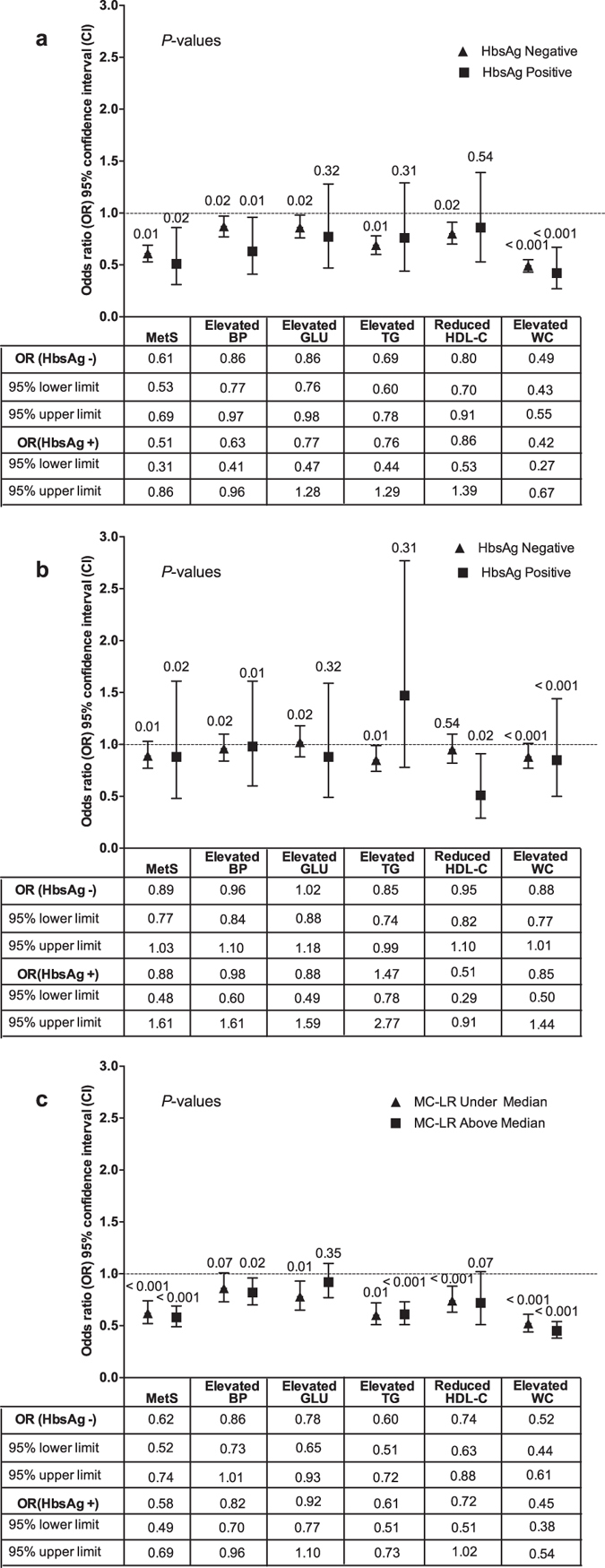
The modification effects of HBV infection on relationships of MetS and its constituents with AFB1 exposure and MC-LR exposure; and the modification effects of MC-LR exposure on relationships of MetS and its constituents with AFB1 exposure. (**a**) The modification effects of HBV infection on ﻿the association between MetS  and Aflatoxin B1 exposure. (**b**) The modification effects of HBV infection on the association between MetS and MC-LR exposure. (**c**) The modification effects of MC-LR exposure on the association between the MetS and Aflatoxin B1 exposure. The higher AFB1 exposure significantly decreased the risk of MetS no matter whether the HBV infection was positive or negative. For people with the higher AFB1 exposure, the number of the constituents of MetS with decreased risk was higher in those without HBV infection than in those with HBV infection. For people either with or without HBV infection, those exposed to the higher or lower MC-LR levels showed no difference in the risk of MetS and all its constituents.

For people without HBV infection, those exposed to the higher AFB1 levels had the significantly decreased risk of MetS (OR = 0.61, 95% CI: 0.53–0.69) and all its constituents, compared with those exposed to the lower AFB1 levels; for people with HBV infection, those exposed to the higher AFB1 levels had the significantly decreased risk of MetS (OR = 0.51, 95% CI: 0.31–0.86), elevated BP (OR = 0.63, 95% CI: 0.41–0.96), elevated WC (OR = 0.42, 95% CI: 0.27–0.67), compared with those exposed to the lower AFB1 levels (Fig. 6a). For people either with or without HBV infection, those exposed to the higher or lower MC-LR levels showed no difference in the risk of MetS and all its constituents (Fig. 6b). For people exposed to the lower MC-LR levels, those also exposed to the higher AFB1 levels had the decreased risk of the risk of MetS (OR = 0.62, 95% CI: 0.52–0.74) and all its constituents except for elevated BP (OR = 0.86, 95% CI: 0.73–1.01), compared with those exposed to the lower AFB1 levels; for people exposed to the higher MC-LR levels, those exposed to the higher AFB1 levels had the decreased risk of MetS (OR = 0.58, 95% CI: 0.49–0.69), elevated BP (OR = 0.82, 95% CI: 0.70–0.96), elevated TG (OR = 0.61, 95% CI: 0.51–0.73), and elevated WC (OR = 0.45, 95% CI: 0.38–0.54), compared with those exposed to the lower AFB1 levels (Fig. 6c).

## Discussion

The prevalence of MetS was higher in rural areas in Southwest China where economy develops relatively slow, and the prevalence of liver disease is higher than that in other provinces in China. We observed that the unadjusted prevalence of the MetS in our study (31.8%) was lower than that reported among adult residents in Chinese urban community (33.9%)^9^ according to 2009 Joint scientific Statement. However, the prevalence in our study was still higher than that in other two reports: In one of these two reports, the prevalence was 24.5% in mainland China by a meta-analysis despite of the different criteria^10^, and in the other report, the prevalence was 11.0% among all adults and 9.4% among rural adults from data obtained from 150 monitoring sites located in 31 provinces, autonomous regions, and municipalities (2010–2012) in China according to the diagnostic criteria of China Diabetes Society (CDS)^21^. CDS are more restrict than the 2009 Joint Scientific Statement.

Some of the lifestyle factors of people living in this rural area were also shown to be associated with the higher prevalence of MetS in this study. The average family income and education levels were not associated with MetS, which might be attributed to the fact that most of the people had lower levels of education (only 6.5% of the people received senior high school education) and average family income (42.8% of the people had a level of income lower than the average income of 6000 RMB/year). Some other known risk factors such as alcohol drinking^22, 23^, salt consuming^24^, and some known protective factors such as fresh fruit and vegetable^25^ intake were also observed in this study, but no association with MetS was found. All those results suggested that most people living in rural areas of Southwest China have almost the same lifestyle. However, we also observed that the risk of MetS increased among people with advanced age, lower labor strength, and fat consumption more than once a week^26^, which is consistent with the findings in the previous reports. Furthermore, we observed that the prevalence in our study was lower in men (19.8%) and higher in women (39.0%)^9^, compared with that in other places (35.0% in men and 27.8% in women). The reason might be that men in rural areas usually do more labor work than women in China: about 33.6% women had lower labor strength but only 28.3% men had lower labor strength (data not shown) in this study. Interestingly, we observed that living in the village was a risk factor for MetS, but the reason might be complicated. In China, people living in small towns usually have mental superiority than those living in villages although they have the same levels of family income and education. It is a pity that we did not collect those data, and then the underlying reason needs further study.

The liver is the key organ for lipoprotein synthesis and gluconeogenesis. Theoretically, Patients with ALF showed an increase in the serum glucose and lipid level, which might make contribution to MetS. Serum liver enzymes, such as ALT, AST, ALP, and GGT, are usually used as surrogate markers of liver function injury. Therefore, we defined it as ALF if two of them are elevated. Several studies have shown the associations between abnormally high levels of liver enzyme and risks of many diseases including MetS. For example, ALT and AST are used as surrogate markers of NAFLD, and they also reflect hepatic expressions of MetS at an early stage^11, 12^; increased levels of ALT in liver are associated with vascular endothelial disorders and body insulin sensitivity, which are independent of obesity^27^; associations of elevated ALT and AST levels with MetS have also been reported^28^. In this study, we observed that ALF was positively associated with MetS and all its constituents (Fig. 3). To our knowledge, many factors including alcohol drinking, fat consumption, hepatitis virus infection, bio-toxin exposure, drug using can damage the liver function. Interestingly, we observed that some risk factors for ALF decreased the risk of MetS, such as HBV infection, higher AFB1 exposure (EDI), and smoking.

HBV infection is another known important public health problem in China, especially to adults over 40 years old without regular hepatitis B vaccination^29^. It has been reported that HBV infection rate among those aged 1–60 years was 7.18% in 2006^30, 31^. It is also well known that chronic HBV infection has an inverse association with all lipid profiles including cholesterol, TG, HDL-C and low-density lipoprotein cholesterol (LDL-C)^32^. Firstly, HBV X protein (HBx), a protein originated from the HBV genome, can cause hepatic lipid deposition by up-regulating the gene expressions of various lipogenic and adipogenic enzymes in hepatic cells^33^. Secondly, HBx can also inhibit apolipoprotein B secretion by enhancing the expression of N-Acetylglucosaminyltransferase III, and then induce the lipid accumulation in hepatic cells^34^, which might lower the serum lipid levels. Thirdly, HBV DNA is found to have a positive correlation with serum adiponectin (antidiabetic and antiatherogenic adipokine)^17^, whose circulating levels are reduced in subjects with diabetes and coronary artery disease. Lastly, HBV infected patients usually have a healthier dietary habit because of health consciousness. In this study, HBV infection had the higher prevalence (8.9%), and it was also a protective factor for MetS. Furthermore, higher HBV DNA titers were a protective factor for MetS and two of its constituents (elevated TG and elevated GLU), but did not change the risk of reduced HDL-C (Fig. 2). This result is contradictory to that in a published study reported by Jarcuska, P. *et al*., who found that HBV patients diagnosed with MetS had higher HBV DNA load than those without MetS, and that patients with TC and apolipoprotein B100 in the reference range had lower HBV DNA load than those with either high or low levels of TC or apolipoprotein B100^35^. In other published studies, some show an inverse relationship, some show a positive relationship, and the others show no association between MetS and HBV infection. However, most HBV infected patients would theoretically progress toward NAFLD in the foreseeable future, and NAFLD is a hepatic expression of MetS at the early stage. Therefore, although HBV infection is a protective factor for MetS in this study, it might also exert risk effects on the disease progression especially during the progression to NAFLD. It is a pity that we did not diagnose NAFLD in this study. More studies should be conducted to explain whether NAFLD is the key process on the way from HBV infection to MetS.

The association between chronic AFB1 exposure and MetS is less reported. However, relationships between the AFB1 exposure and serum lipid level were contradictory. Some research studies found that AFB1 can significantly decrease the serum triglycerides^36^, phospholipids and cholesterol, but increase the lipids that accumulate in the liver by reducing fat oxidation or stimulating lipid synthesis^37, 38^. Another research reported that the serum glucose and cholesterol level of rats are elevated after exposure to AFB1 (1000 ppb) in diets^39^. In this study, the inverse association between the AFB1 exposure (EDI) and MetS was observed, and the inverse association between the AFB1 and hyperglycemia (OR = 0.84, 95% CI: 0.75–0.95) was also significant.

The association between chronic MC-LR exposure and MetS is also less reported. Mice exposed to 20 mg/kg of MC-LR had disrupted glucose, triglyceride and cholesterol metabolism with obvious weight loss^40^, which might be related to the endoplasmic reticulum stress, but the mechanism remains unclear. In this study, the association between the MC-LR exposure (EDI) and MetS was not significant, which might be attributed to the lower level of MC-LR exposure (median 3.17 ng/day/kg).

In this study, HBV infection and the higher AFB1 exposure (EDI) were inversely associated with MetS no matter whether the liver function was normal or abnormal (Fig. 5). However, for people with HBV infection, the number of the constituents of MetS with decreased risk was higher in those showing abnormal liver function than in those showing normal liver function (Fig. 5a). For people with the higher AFB1 level, the number of the constituents of MetS with decreased risk were lower in those showing abnormal liver function than in those showing normal liver function (Fig. 5b). All those results suggested that the liver function might not decide the progression but the severity of MetS. Furthermore, the higher AFB1 exposure significantly decreased the risk of MetS no matter whether the HBV infection was positive or negative (Fig. 6a). However, for people with the higher AFB1 exposure, the number of the constituents of MetS with decreased risk was higher in those without HBV infection than in those with HBV infection. This is very interesting because HBV infection and higher AFB1 exposure can decrease the risk of all its constituents. Therefore, there might be an interaction effect between the higher AFB1 exposure and HBV infection, but the mechanism needs to be further studied.

To sum up, MetS has the higher prevalence, especially among women in rural areas located in Southwest China, although the economy there develops slower and the education level of the residents are lower than those in other areas. The risk factors of lifestyle for MetS in this area is lower labor strength and consuming fat more than once a week. The ALF showed as a risk factor for MetS. The HBV infection and higher AFB1 exposure (EDI) showed a protective effect on MetS, but higher MC-LR exposure (EDI) showed no effect on MetS in this study. Therefore, more attention must be paid to the people with HBV infection, higher AFB1 exposure (EDI) and ALF, to prevent MetS and to decrease its prevalence. Whether HBV infection and AFB1 exposure became the risk factor for MetS on the disease progression to NAFLD should be confirmed in the future by well-designed and longitudinal follow-up studies.

## Materials and Methods

### Study population and ethics statement

Subjects were from a population-based cross-sectional survey on hepatocellular carcinoma in two towns (Lidu and Yihe) of the Fuling District, Chongqing, situated at the Three Gorge Reservoir Region, Southwest China. Briefly, from June to July in 2013, 6467 people aged from 30 to 85 years old and having lived in Lidu and Yihe for at least 10 years were recruited in the study. Among them, 5493 individuals (2067 men and 3426 woman) who completed the questionnaires and clinical examinations were included in the study^20^.

This study was approved by the Ethics Committee of the Third Military Medical University and the Center for Disease Prevention and Control (CDC) in Fuling District. Written informed consent for participation was obtained from each participant. All methods were performed in accordance with the relevant guidelines and regulations.

### Anthropometric and laborites measurements

The questionnaire was used to collect information of smoking status, alcohol consumption, education level, labor intensity, family income, and fat consumption. A current smoker was defined as someone who had smoked at least one cigarette per day for more than 6 months; a drinker was defined as someone who had drunk any kind of alcohol at least once a week for more than 6 months. The daily consumption amounts of local drinking water (L/daily) and aquatic products (fish and duck) (g/daily) suspected to be contaminated by microcystin and local food (g/daily) suspected to be contaminated by aflatoxin were also recorded. The data collectors, who were trained with ethical principles and by standard methods, filled in the questionnaire directly as soon as they interviewed the subjects face to face.

Body weight, height, waist circumference (WC) and blood pressure were measured by physicians according to standard methods^41^. WC was measured at the midpoint between the lower margin of the least palpable rib and the top of the iliac crest^42^. Blood pressure was measured in left arm at heart level after the subject seated with their legs resting on the ground for 5 minutes^43^.

A venipuncture system was used by trained nurses to collect blood samples from subjects in the morning prior to their first meal. Blood samples were tested for HBV infection, blood lipids, glucose and liver function. HBsAg (enzyme immunoassay) in serum was measured by using commercial kits (Dainabot Co. Ltd., Tokyo, Japan). HDL-cholesterol and triglycerides (TG) were detected by biochemical automatic analyzer (Hitachi 7600). Alanine aminotransferase (ALT), aspartate aminotransferase (AST), alkaline phosphatase (ALP) and gamma glut amyl transferase (GGT) were assayed by using Synchron Clinical System LX20 (Beckman-Coulter Diagnosis, Fullerton, CA, USA). ALF was defined as when more than two serum enzymes are above the Upper Limit of Normal as previously described^44^.

### Assessment of microcystin and aflatoxin exposure

The assessment of microcystin and aflatoxin exposure was performed according to the methods previously described^20, 44^. Briefly, 5 L of eight types of source water and drinking water, and five types of carp were collected once a month for microcystin-LR analysis. In the meantime, 13 types of daily food were collected from local residents and purchased in the local markets for AFB1 analysis. Microcystin-LR and AFB1 were assessed by using the ELISA method. Individual toxin exposure level was measured based on EDI that was calculated according to the microcystin-LR levels in water and aquatic products and AFB1 levels in daily food.

### Definition of MetS

According to the 2009 Joint scientific Statement^1^, the presence of any three of the following five risk factors constitutes a diagnosis of metabolic syndrome, including waist circumference (man ≥85 cm and women ≥80 cm), triglycerides ≥150 mg/dL (1.7 mmol/L), HDL-C <40 mg/dL (1.0 mmol/L) in males or <50 mg/dL (1.3 mmol/L) in females, systolic ≥130 and/or diastolic ≥85 mm Hg, fasting glucose ≥100 mg/dL or drug treatment as an alternate indicator.

### Statistical analysis

All data were analyzed with SPSS20.0. In the initial data analysis, ﻿medians﻿ of levels of MC-LR and Aflatoxin B1 were used as cutoff points. Data were presented as mean ± standard deviation (SD) or median (interquartile range) as appropriate. Chi-square was used to examine the difference between men and women. Binary logistic regression analyses were used to determine odds ratios (ORs) and 95% confidence intervals (CIs) of the HBV infection, MC-LR exposure and Aflatoxin B1 exposure  to MetS. We also performed analyses of other potential risk factors including place of residence (small town, village), sex (male, female), age, smoking status (never, ever), alcohol drinking (never, ever), fat consumption (never, once a week, above once a week), salt consumption (recommended daily intake - RDI, less, more), fresh fruit and vegetable intake (RDI, less, more), liver function (normal, abnormal), HBV infection (HbsAg negative, HbsAg positive), MC-LR exposure (below median, above median), AFB1 exposure (below median, above median), labor strength (appropriate, low, heavy), family income (average, less, more), and education level (primary school, junior high school). All the statistical analyses were conducted by using SPSS 20.0 and *P* value less than 0.05 was considered significant (2-sided).

## References

[1] Alberti KG (2009). Harmonizing the metabolic syndrome: a joint interim statement of the International Diabetes Federation Task Force on Epidemiology and Prevention; National Heart, Lung, and Blood Institute; American Heart Association; World Heart Federation; International Atherosclerosis Society; and International Association for the Study of Obesity. Circulation.

[2] Isomaa B (2001). Cardiovascular morbidity and mortality associated with the metabolic syndrome. Diabetes Care.

[3] Salminen M (2013). Metabolic syndrome defined by modified International Diabetes Federation criteria and type 2 diabetes mellitus risk: a 9-year follow-up among the aged in Finland. Diab Vasc Dis Res.

[4] Eckel RH, Grundy SM, Zimmet PZ (2005). The metabolic syndrome. The Lancet.

[5] Kolovou GD, Anagnostopoulou KK, Salpea KD, Mikhailidis DP (2007). The prevalence of metabolic syndrome in various populations. Am J Med Sci.

[6] Scuteri A (2015). Metabolic syndrome across Europe: Different clusters of risk factors. European Journal of Preventive Cardiology.

[7] Ford ES, Li CY, Zhao GX (2010). Prevalence and correlates of metabolic syndrome based on a harmonious definition among adults in the US. J Diabetes.

[8] He Y (2013). Dietary patterns as compared with physical activity in relation to metabolic syndrome among Chinese adults. Nutr Metab Cardiovas.

[9] Wang, G. R. *et al*. Prevalence of metabolic syndrome among urban community residents in China. *Bmc Public Health* **13**, doi:Artn 59910.1186/1471-2458-13-599 (2013). 10.1186/1471-2458-13-599 PMC3734094 23786855

[10] Li R (2016). Prevalence of metabolic syndrome in Mainland China: a meta-analysis of published studies. BMC Public Health.

[11] Fan JG, Farrell GC (2009). Epidemiology of non-alcoholic fatty liver disease in China. J Hepatol.

[12] Pagano G (2002). Nonalcoholic steatohepatitis, insulin resistance, and metabolic syndrome: further evidence for an etiologic association. Hepatology.

[13] Jung KY, Cho SY, Kim HJ, Kim SB, Song IH (2014). Nonalcoholic steatohepatitis associated with metabolic syndrome: relationship to insulin resistance and liver histology. J Clin Gastroenterol.

[14] Suhail M (2014). Potential mechanisms of hepatitis B virus induced liver injury. World journal of gastroenterology.

[15] Yotsuyanagi H (2002). Precore and core promoter mutations, hepatitis B virus DNA levels and progressive liver injury in chronic hepatitis B. J Hepatol.

[16] Li WC (2013). Association between the hepatitis B and C viruses and metabolic diseases in patients stratified by age. Liver Int.

[17] Hsu CS (2012). Impact of hepatitis B virus infection on metabolic profiles and modifying factors. J Viral Hepat.

[18] Chung TH, Kim MC, Kim CS (2014). Association between Hepatitis B Surface Antigen Seropositivity and Metabolic Syndrome. Korean J Fam Med.

[19] Jan CF (2006). A population-based study investigating the association between metabolic syndrome and hepatitis B/C infection (Keelung Community-based Integrated Screening Study No. 10). Int J Obesity.

[20] Lin H (2016). Determination of Environmental Exposure to Microcystin and Aflatoxin as a Risk for Renal Function Based on 5493 Rural People in Southwest China. Environ Sci Technol.

[21] He YN (2017). [Prevalence of metabolic syndrome in Chinese adults in 2010–2012]. Zhonghua Liu Xing Bing Xue Za Zhi.

[22] Xiao J (2015). Association of alcohol consumption and components of metabolic syndrome among people in rural China. Nutr Metab (Lond).

[23] Slagter SN (2014). Combined effects of smoking and alcohol on metabolic syndrome: the LifeLines cohort study. PLoS One.

[24] Oh SW (2015). Association of Sodium Excretion With Metabolic Syndrome, Insulin Resistance, and Body Fat. Medicine (Baltimore).

[25] Cheraghi Z (2016). The association between nutritional exposures and metabolic syndrome in the Tehran Lipid and Glucose Study (TLGS): a cohort study. Public Health.

[26] Narasimhan S (2016). Dietary fat intake and its association with risk of selected components of the metabolic syndrome among rural South Indians. Indian J Endocrinol Metab.

[27] Ludwig J, McGill DB, Lindor KD (1997). Review: nonalcoholic steatohepatitis. J Gastroenterol Hepatol.

[28] Scuteri A (2015). Metabolic syndrome across Europe: different clusters of risk factors. Eur J Prev Cardiol.

[29] Liang P (2015). The independent impact of newborn hepatitis B vaccination on reducing HBV prevalence in China, 1992–2006: A mathematical model analysis. J Theor Biol.

[30] Cui Y, Jia J (2013). Update on epidemiology of hepatitis B and C in China. J Gastroenterol Hepatol.

[31] Liang X (2013). Reprint of: Epidemiological serosurvey of Hepatitis B in China–declining HBV prevalence due to Hepatitis B vaccination. Vaccine.

[32] Jan CF (2006). A population-based study investigating the association between metabolic syndrome and hepatitis B/C infection (Keelung Community-based Integrated Screening study No. 10). Int J Obes (Lond).

[33] Kim KH (2007). Hepatitis B virus X protein induces hepatic steatosis via transcriptional activation of SREBP1 and PPARgamma. Gastroenterology.

[34] Kang SK (2004). The hepatitis B virus X protein inhibits secretion of apolipoprotein B by enhancing the expression of N-acetylglucosaminyltransferase III. J Biol Chem.

[35] Jarcuska P (2014). Hepatitis B virus infection in patients with metabolic syndrome: a complicated relationship. Results of a population based study. Eur J Intern Med.

[36] Amiridumari H, Sarir H, Afzali N, Fanimakki O (2013). Effects of milk thistle seed against aflatoxin B1 in broiler model. J Res Med Sci.

[37] Zhang L (2011). Systems responses of rats to aflatoxin B1 exposure revealed with metabonomic changes in multiple biological matrices. J Proteome Res.

[38] Siloto EV (2013). Lipid metabolism of commercial layers fed diets containing aflatoxin, fumonisin, and a binder. Poult Sci.

[39] Abdel-Latif, M. S., Elmeleigy, K. M., Aly, T. A., Khattab, M. S. & Mohamed, S. M. Pathological and biochemical evaluation of coumarin and chlorophyllin against aflatoxicosis in rat. *Exp Toxicol Pathol*, doi:10.1016/j.etp.2017.01.014 (2017). 10.1016/j.etp.2017.01.014 28190563

[40] Zhao Y (2015). Microcystin-LR induced thyroid dysfunction and metabolic disorders in mice. Toxicology.

[41] Pickering TG (2005). Recommendations for blood pressure measurement in humans and experimental animals: part 1: blood pressure measurement in humans: a statement for professionals from the Subcommittee of Professional and Public Education of the American Heart Association Council on High Blood Pressure Research. Circulation.

[42] WHO. Waist circumference and waist–hip ratio: report of a WHO expert consultation, Geneva, 8–11. *WHO Library Cataloguing-in-Publication Data*, 1–7 (2008).

[43] Weber MA (2014). Clinical practice guidelines for the management of hypertension in the community: a statement by the American Society of Hypertension and the International Society of Hypertension. J Clin Hypertens (Greenwich).

[44] Li Y (2011). A cross-sectional investigation of chronic exposure to microcystin in relationship to childhood liver damage in the Three Gorges Reservoir Region, China. Environ Health Perspect.

